# Supplementary material for: Preparation and Characterization of Polyethersulfone-Ultrafiltration Membrane Blended with Terbium-Doped Cerium Magnesium Aluminate: Analysis of Fouling Behavior
Source: Molecules. 2023 Mar 16;28(6):2688. doi: 10.3390/molecules28062688 (PMC10051232; doi:10.3390/molecules28062688)
Supplement: Supplementary file 1 [file molecules-28-02688-s001.zip › molecules-2184983-supplementary.pdf]

Supplementary Information

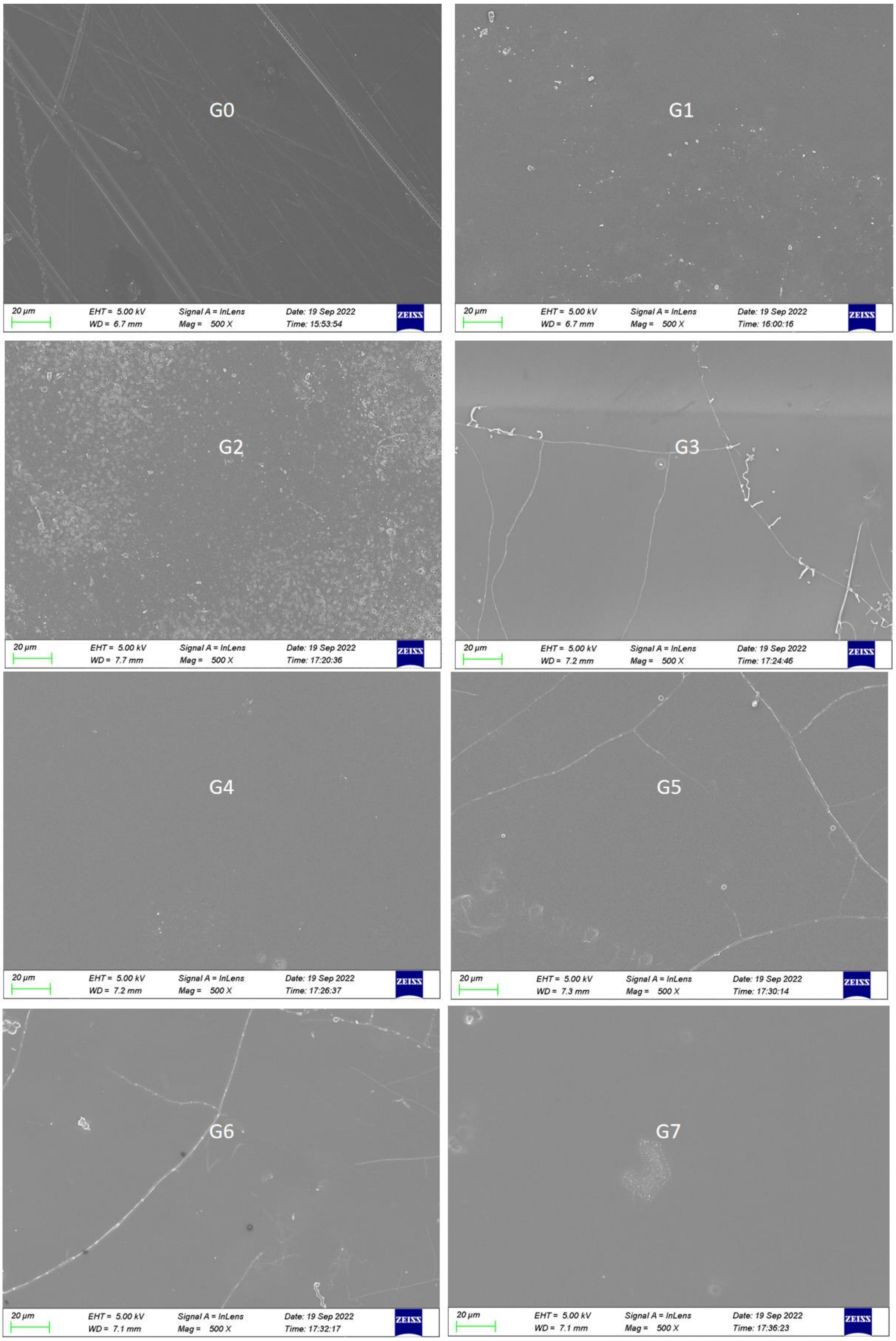

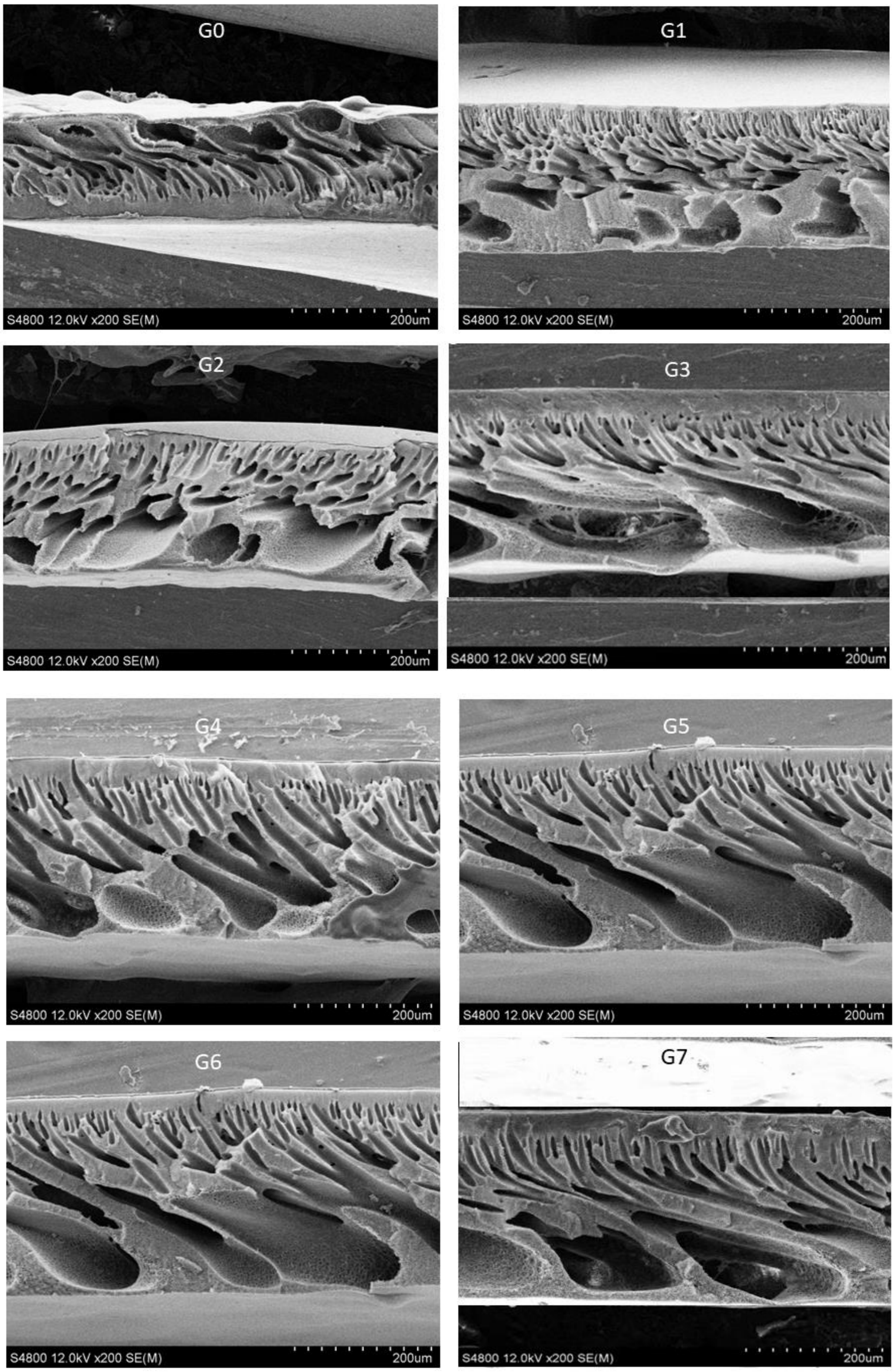

Figure S1: SEM images of the top layer and cross-sectional surface morphology.

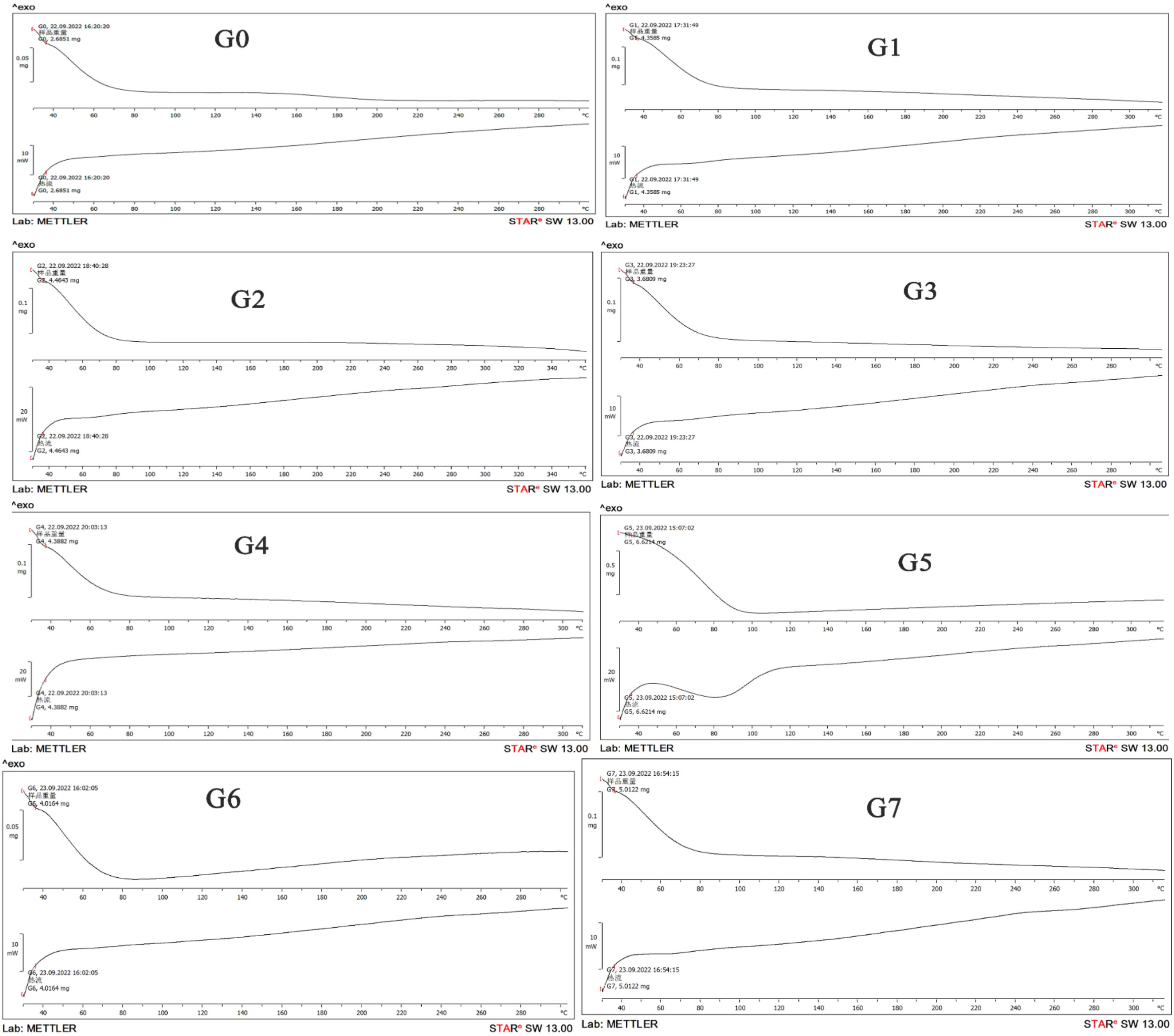

Figure S2: TGA curves of the raw PES origin membrane G0 and the modified PES membranes from G1 to G7.
